# Transparent Elastic Wound Dressing Gel Supporting Drug Release: Synergistic Effects of Poly(Vinyl Alcohol)/Chitosan Hybrid Matrix

**DOI:** 10.3390/gels11100771

**Published:** 2025-09-25

**Authors:** Lifei Chen, Ningning Yuan, Zhenjiang Tan, Jianwei Zhang, Lishi Zhang, Wenwei Tang, Cheng Chen, Donghai Lin

**Affiliations:** 1 Shanghai Key Laboratory of Engineering Materials Application and Evaluation, School of Energy and Materials, Shanghai Polytechnic University, Shanghai 201209, China; 2 Shanghai Thermophysical Properties Big Data Professional Technical Service Platform, Shanghai Engineering Research Center of Advanced Thermal Functional Materials, Shanghai 201209, China; 3 School of Resources and Environmental Engineering, Shanghai Polytechnic University, Shanghai 201209, China; 4 School of Mathematics Physics and Statistics, Shanghai Polytechnic University, Shanghai 201209, China

**Keywords:** nanosilver, wound healing, antimicrobial, dressing

## Abstract

Wound infection is one of the most critical factors affecting the healing process. Therefore, the development of wound dressings with excellent antibacterial effects has become a research hotspot in the current academic field. We prepared AgNPs (silver nanoparticles) via a redox method, combined them with Poly(vinyl alcohol)/chitosan (PVA/CS), and dried the mixture into a film to fabricate a silver-loaded hydrogel film dressing with excellent antibacterial properties. Uniaxial tensile tests on the samples revealed that the prepared film dressings exhibited good mechanical properties, preventing fracture caused by external forces. Protein adsorption experiments indicated their favorable protein adsorption performance, which can adsorb microorganisms on the external surface of the dressing. By leveraging the bactericidal mechanism of AgNPs, the dressing achieves efficient antibacterial effects. Additionally, the dressing prepared by this method features good transparency, facilitating routine observation of the wound area without removing the dressing and maintaining a sterile environment for an extended period. Finally, we verified the drug loading and drug release capabilities of the dressing, and found that it has good drug loading capacity and drug release effect. This preliminarily proves its effectiveness and provides more possibilities for subsequent research on composite drugs. This study provides new insights for exploring the clinical application of multifunctional silver-loaded wound dressings.

## 1. Introduction

Wound infection is one of the most important factors affecting the healing process and may even lead to the formation of chronic wounds [1]. Bacterial contamination of skin wounds, on the other hand, is the main cause of sepsis, which is associated with high morbidity and mortality. Therefore, it is important to develop wound dressings with efficient antimicrobial properties to protect against bacterial infections during the wound healing process. In recent years, a variety of novel multifunctional hydrogel wound dressings have been designed to absorb wound exudate and promote wound healing during the treatment of burns, traumas, and various chronic wounds [2,3]. Ideal wound dressings should achieve rapid wound healing to minimize potential patient suffering, but conventional wound dressings, such as gauze and taping, only serve to protect the wound from contamination and do not actively participate in the wound healing process [4,5]. More advanced new multifunctional dressings are designed to be biologically active on their own or to release biologically active components in the dressing, such as the addition of antimicrobial agents, which can aid in tissue regeneration and accelerate wound healing [6,7].

However, the misuse of antibiotics has led to the gradual development of resistance to some organic antimicrobial agents, thus limiting their clinical application [8]. Inorganic antimicrobial agents have gradually received widespread attention in biomedical applications due to their broad-spectrum antimicrobial properties, low drug resistance, and long-lasting antimicrobial effects. Among the known inorganic nanoparticles, silver nanoparticles (AgNPs) not only have antimicrobial ability against a wide range of bacteria (e.g., Gram-positive, Gram-negative, etc.), but also take a short time to sterilize and have a fast speed, leading to the fact that the antimicrobial properties of AgNPs are not susceptible to drug resistance [9,10,11]. According to research, when using Ag^+^ as an antimicrobial agent for wound dressings, the released Ag^+^ will combine with proteins in plasma or react with Cl^−^ in the body to precipitate, leading to a decrease in its antimicrobial performance. Compared with the ionic form, AgNPs exist in the form of Ag^0^, which is not easy to combine with halide, effectively solving the problem that the rapid inactivation of Ag^+^ cannot be sustained antimicrobially, as well as having a superior antimicrobial effect [12]. The antimicrobial mechanism of AgNPs is mainly based on silver ion release, cell membrane damage (AgNPs are deposited on the bacterial membrane through electrostatic interactions, damaging the cell) [13], DNA interactions (AgNPs contribute to the accumulation of reactive oxygen species (ROS) that activate DNA to damage bacterial cystatin-like proteins) [14], and free radical production [15]. Therefore, combining AgNPs with efficient antimicrobial properties with various polymers has become a versatile strategy to improve the antimicrobial properties of polymers.

In the current work, sodium borohydride was used as a reducing agent to reduce Ag^+^ to Ag^0^ in silver nitrate, while sodium citrate (SC) was used as a stabilizer to solve the problem of the poor stability of AgNPs in solution. Based on previous studies, a novel multifunctional silver-loaded hydrogel film dressing was prepared by combining the synthesized AgNPs with a PVA/CS matrix, and exhibited excellent antimicrobial properties. In addition, the porous structure of the PVA/CS hydrogel provided the prepared silver-loaded hydrogel film dressing with good swelling and mechanical properties. Therefore, the prepared transparent dressing with elastic, non-adhesive properties helps to observe the wound area routinely without removing the dressing and maintaining a sterile environment for a longer period. Drug loading and drug release experiments showed that the dressing can load drugs efficiently and release them. The drug release amount and complete drug release time of dressings with different silver nanoparticle contents varied under different pH environments, showing pH-responsive characteristics. Compared with traditional wound dressings, the film dressing in this study improves transparency while ensuring good swelling capacity, allowing for the observation of wound healing without removing the dressing. Additionally, many previous studies lacked experiments on drug loading and drug release capabilities. By verifying the excellent drug loading and release capabilities of the proposed dressing, this study demonstrates its practical value.

## 2. Results and Discussion

### 2.1. Analysis of Particle Size and Zeta Potential of Silver Nanoparticles

**Figure 1:**
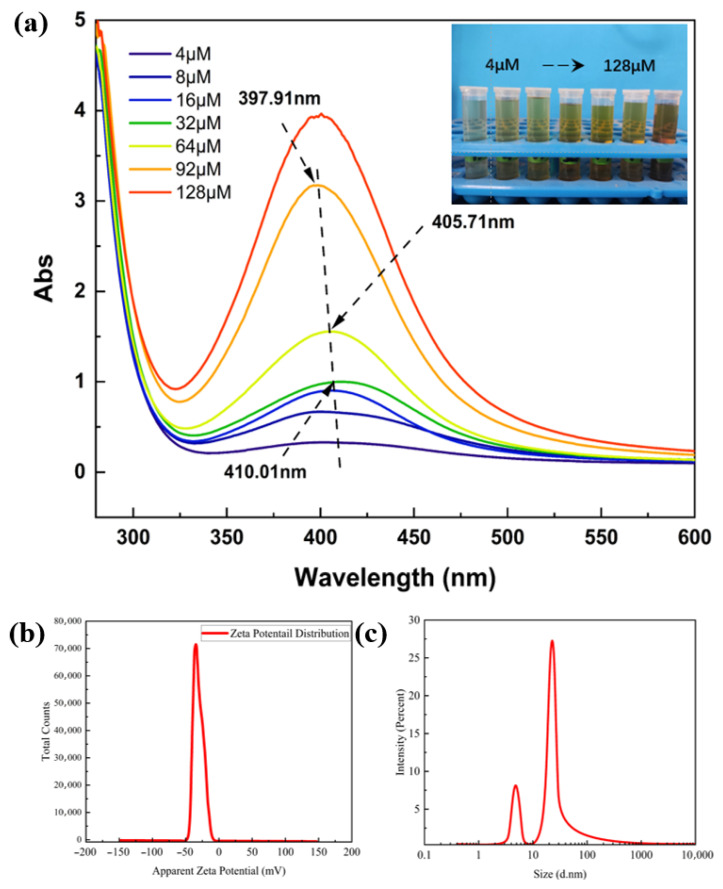
(**a**) UV–Vis absorption spectra of AgNP sols reduced using NaBH_4_ solutions with different concentration gradients; (**b**) particle size and (**c**) zeta potential test plots of AgNP sols.

The UV–Vis absorption spectra of AgNPs can qualitatively reflect the content of nanoparticles (the higher the intensity of the absorption peak, the higher the content of nanoparticles) and particle size (the larger the nanoparticles, the stronger the aggregation, the more positive the total charge of the metal clusters, and the more obvious the red shift) [16,17]. Therefore, a UV–Vis spectrophotometer was used to determine the UV–Vis absorption spectra of AgNP sols reduced from different concentrations of NaBH_4_ solution. As shown in Figure 1a, the UV absorption spectra of the synthesized sols were significantly different for different concentrations of the NaBH_4_ solution.

As the concentration of NaBH_4_ solution increases, the intensity of the absorption peak increases and a blueshift phenomenon occurs. The wavelength of the absorption peak blueshifted to 405.71 nm at a concentration of 64 µM, which indicates that increasing the concentration of NaBH_4_ solution is favorable for the generation of more nanosilver, while also leading to the reduction in the particle size of the nanosilver. To find the critical value, the concentration of NaBH_4_ solution was continuously increased; at 92 µM, the absorption peak wavelength was 397.91 nm, but when the concentration of NaBH_4_ solution was increased to 128 µM, it was found that the position of the absorption peaks remained unchanged and an instantaneous distortion occurred. In summary, NaBH_4_ solution with a concentration of 92 µM was chosen to be used for the reduction in AgNP sols. The synthesized AgNP sols were tested for particle size and zeta potential, as shown in Figure 1b. The particle size range of silver nanoparticles was concentrated at 22 nm and 5 nm. Compared with the study by Zhang et al. [18], the silver nanoparticles prepared by this method have a smaller particle size. The small particle size of silver nanoparticles provides a necessary condition for excellent antimicrobial activity [19]. Therefore, the particle size of the prepared silver nanoparticles is suitable for application in wound dressings. The zeta potential test revealed the presence of a negative charge distribution on the surface of the silver nanoparticle colloid at room temperature, with a potential value of −30.9 mV; therefore, the AgNP sol exhibited good stability.

### 2.2. Characterization of Silver-Loaded Film Dressing Samples

**Figure 2:**
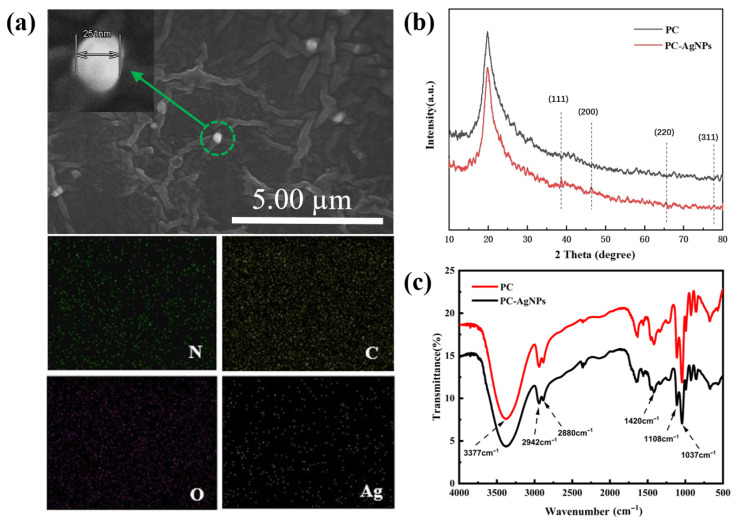
(**a**) SEM-EDS images of elemental distributions of C, O, N, and Ag elements in thin film samples loaded with AgNPs; (**b**) XRD patterns of PC, PC-AgNPs; (**c**) FTIR patterns of PC and PC-AgNPs.

As shown in the SEM image of Figure 2a, the prepared silver-loaded film dressing samples have a porous mesh structure, and the interconnected pores can realize the exchange of gases and nutrients, and the circulation of biologically active molecules, which can effectively promote the proliferation of the cells and ensure their ability to absorb the wound exudate [20,21]. Meanwhile, it can be observed that the synthesized silver nanoparticles were successfully loaded in the PVA/CS hydrogel network structure and were mostly uniformly spherical. The increase in the size of silver nanoparticles is attributed to the increase in the viscosity of the polymer system that hinders the diffusion of nanoparticles, resulting in some agglomeration of AgNPs in the PVA/CS solution [22]. In order to determine the distribution of silver nanoparticles in the PVA/CS hydrogel film, it was characterized by elemental mapping. Figure 2a reveals that Ag, the characteristic element of the silver nanoparticles, and the other basic elements are uniformly distributed throughout the entire sampling range, which means that the elements are in a uniformly distributed state throughout the film samples, indicating that the silver nanoparticles are in the process of high-speed mixing. The silver nanoparticles are fully mixed and have good dispersion in the film sample. PC refers to the sample without AgNPs added. The sample PC-AgNPs used in the sample characterization section is identical to the PC-Ag3 mentioned in the following.

As shown in Figure 2b, due to the addition of trace amounts of AgNP sol, the proportion of silver in the thin film samples is low; therefore, the XRD patterns show diffraction peaks with relatively weak peak intensities. However, it can still be seen that the thin film samples loaded with nanosilver show four different diffraction peaks near the 2θ angles of 38.74°, 46.39°, 65.55°, and 77.74°, corresponding to the standard (111), (200), (220), and (311) crystal planes of the crystalline silver.

The IR spectra of PC and PC-Ag are shown in Figure 2c. The peaks at 3750~3086 cm^−1^ for the PC film sample are generated by O-H and N-H stretching vibrations, which are characteristic peaks of PVA. While the two peaks observed at 2942 cm^−1^ and 2880 cm^−1^ are due to the asymmetric stretching of -CH_2_ and the C-H stretching of CS and PVA chains, the absorption peaks at 1037 cm^−1^ and 1420 cm^−1^ are due to C-O stretching and C-H bending. Compared with the control PC film samples, the positions of the absorption peaks of the AgNP-treated film samples remained basically unchanged and no new characteristic peaks appeared, suggesting that the addition of AgNPs did not form new chemical bonds. However, it can be observed that, after the addition of nanosilver, the overall peak intensity of the infrared spectrogram of the samples tends to weaken, which proves that there is no chemical interaction in the polymer structure during the treatment of AgNPs, but only the physical adsorption and encapsulation of AgNPs in the PVA/CS hydrogel network. But at this time, it will lead to a change in the overall concentration of the solution, and thus a weakening of the peak intensity. This it also indicates that, side by side, AgNPs are well bound to the aqueous gels, with other components of the hydrogel also being well bound, which was proved similarly elsewhere [23].

### 2.3. Mechanical Properties

**Figure 3:**
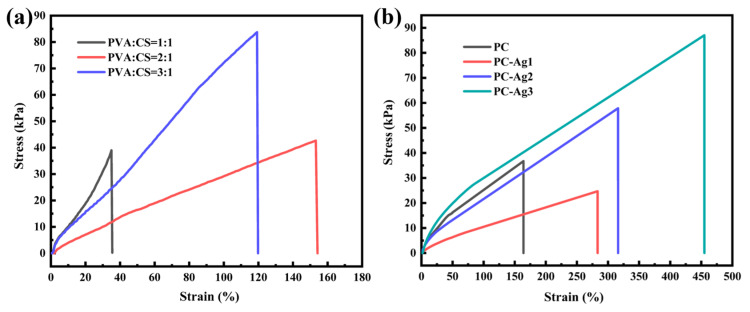
(**a**) Tensile stress–strain curves of film samples with PVA/CS ratios of 1:1, 2:1, and 3:1, respectively; (**b**) tensile stress–strain curves of film samples with different AgNP contents.

Excellent mechanical properties ensure that the film dressing will not break easily during use. While PVA can give hydrogels good mechanical properties, pure PVA hydrogel films are too hard and not tough enough for the human body. According to research, the addition of CS can increase the pore size of PVA/CS hydrogel and reduce the tightness of the hydrogel network structure, which leads to the structure of the hydrogel becoming loose and easier to stretch, thus giving it certain tensile properties. However, too much PVA content will increase the fracture strength, but increase the hardness of the film samples and reduce their tensile properties. Therefore, it is necessary to balance the tensile properties and fracture strength to choose the appropriate ratio of PVA and CS. The experiments were carried out using a universal material tensile testing machine to conduct unidirectional tensile tests on different ratios of PVA/CS hydrogel film samples (1:1, 2:1, and 3:1 for PVA/CS, respectively). As shown in Figure 3a, when PVA/CS = 1:1, the film samples will present a relatively brittle state due to the high content of CS in the whole polymer system; at this time, the tensile rate does not even reach 40%, and the tensile properties are extremely weak and very easy to fracture. When the proportion of PVA is increased, the hydrogel film has better flexibility, the tensile rate reaches 157%, the tensile properties have been greatly improved, and, at the same time, the fracture strength is also gradually balanced. However, by continuously increasing the proportion of PVA, a significant increase in the breaking strength is observed, which limits the tensile properties due to the increased hardness of the film samples at this point. In conclusion, to balance the mechanical strength and tensile properties, PVA/CS = 2:1 is the most suitable ratio. The modulus of elasticity of the skin refers to the elastic properties of the skin when it resists external pressure.

According to the study, the modulus of elasticity of adult skin is about 1.7 MPa. In order to avoid damage to human skin tissues by the prepared dressing samples, the fracture stress of the samples should not be outside the tolerance range of human skin [24]. During the tensile deformation of PVA/CS polymer chains, AgNPs alter their structural rearrangement by promoting semi-crystalline mechanical properties, and nanoparticles with high volume-to-surface ratios (i.e., short inter-particle distances) are loaded onto the polymer chains, resulting in a decrease in the grain size in the films, which improves the material’s plastic deformability and toughness, as well as its mechanical properties [25]. Therefore, based on the PVA/CS ratio of 2:1, the effect of different contents of silver nanoparticles on the mechanical properties of the films was investigated. The sample with 0.5 mL of silver nanoparticle solution added was named PC-Ag1, the sample with 1 mL of silver nanoparticle solution added was named PC-Ag2, and the sample with 1.5 mL of silver nanoparticle solution added was named PC-Ag3. As shown in Figure 3b, unidirectional tensile tests were conducted on film samples loaded with different contents of AgNPs. It was found that the film samples loaded with AgNPs exhibited higher fracture stress and elongation; this was due to the fact that the AgNPs were able to form a closer interfacial bond with the PVA and CS molecules to enhance their strength and toughness, and to fill the microscopic voids and defects of the films so to effectively resist the external stresses and improve the tensile properties. In addition, similar findings were reported separately by Ali Alipour [26] and Wang [27].

### 2.4. Swelling Rate and Porosity

Dr. Winter’s theory of wet wound healing states that, in a humid environment that maintains the fluid balance of the wound, the rate of cellular tissue migration and proliferation is significantly enhanced, resulting in a two-fold increase in the rate of wound healing compared to a completely dry environment [28]. In addition to promoting wound healing, a wet environment can have the ability to autolyze and clear wounds, relieve pain, and promote the migration of new keratinocytes [29,30]. The main function of hydrogel wound dressings is to absorb exudate from open wounds due to their porous structure avoiding infection of normal tissues around the wound. Therefore, good swelling properties are one of the decisive factors for whether the developed wound dressing has a promising market application. In addition, porosity and water contact angle are two important factors reflecting swelling properties, and higher porosity and better hydrophilicity usually imply higher water absorption and air permeability [31]. Therefore, experiments were conducted to test and analyze the porosity, hydrophilicity, and swelling properties of the film samples.

**Figure 4:**
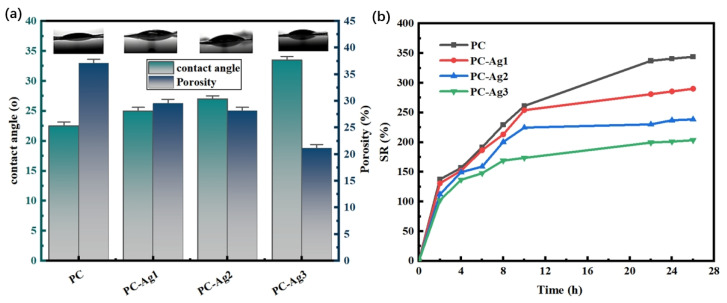
(**a**) Water contact angle and porosity and (**b**) dissolution rate for PC, PC-Ag1, PC-Ag2, and PC-Ag3.

As shown in Figure 4a, the PC film sample has a higher porosity compared to the silver-loaded film sample With the increase in the amount of AgNPs added, more and more small-sized nanoparticles occupy the pores of the hydrogel film, leading to a decrease in the porosity of the hydrogel; at the same time, it can also effectively resist the invasion of external microorganisms and avoid wound infection. Due to the presence of a large number of hydrophilic hydroxyl groups, PVA is a superhydrophilic material with high water absorption [32]. Therefore, the PC film samples have excellent hydrophilic properties. The addition of AgNPs increases the roughness of the film surface, which leads to a gradual increase in the water contact angle. However, due to the limited amount of added AgNPs, the hydrophilicity of the material is less affected, and the water contact angles of the film samples loaded with AgNPs are all below 35°. Overall, the prepared hydrogel films still have good hydrophilicity, which is favorable for the absorption of wound exudate. The porosity of the hydrogel network plays an important role in regulating its swelling behavior. As shown in Figure 4b, the PC film sample had a good swelling rate of 340% after 24 h. However, with the increase in the amount of added AgNPs, the swelling performance of the silver-loaded hydrogel film sample was found to be gradually weakened, which may be due to the fact that the film’s porosity was originally small; moreover, more and more nanoparticles of a small particle size occupied the pores of the hydrogel film. This may be due to the small porosity of the hydrogel films. This also leads to the weakening of the swelling performance of the hydrogel films; however, the swelling rate of PC-Ag3, which is the lowest, can still reach 200%, which proves that the silver-loaded thin film samples still have good swelling performance.

### 2.5. Protein Adsorption Properties

**Figure 5:**
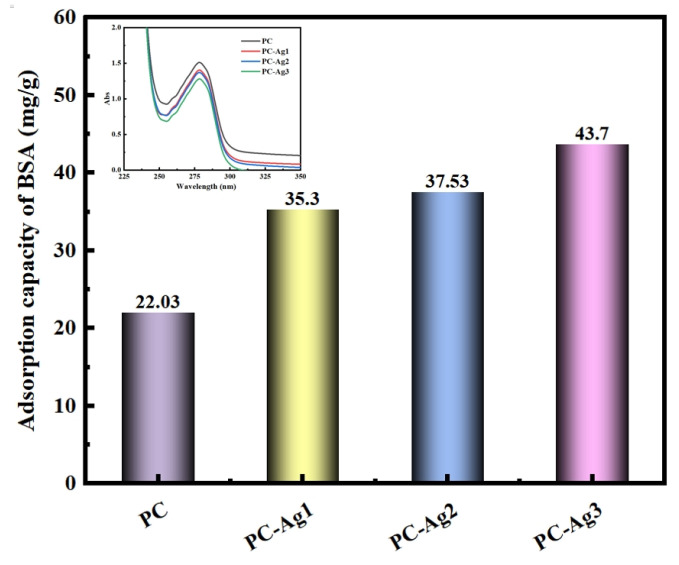
Protein adsorption performance of PC, PC-Ag1, PC-Ag2, and PC-Ag3 samples after being immersed in BSA solution for 24 h.

Bacterial infection is an important factor in wound severity. Airborne microorganisms can contact the wound through the pores of the dressing, leading to wound infection. Since the absorption value of the solution is positively related to the protein concentration, it can be seen from the inset that the PC film samples have higher absorbance compared to the silver-carrying film samples. This proves that, with the higher the concentration of the remaining protein in the solution, the adsorption amount of the PC film samples to the BSA is relatively less, at only 22.03 mg/g. In addition, with the increase in the content of the AgNPs, the absorbance of the solution shows a gradual decrease in the trend, with the protein adsorption capacity then gradually increasing, as shown in Figure 5. It has been reported in the literature that cations can promote dressing–protein interactions, thereby increasing protein adsorption [33]. Silver ions can release divalent cations continuously through the ionization balance, and the electrostatic repulsion of positive charge can prevent the particles from aggregating, and ensure their uniformity and distribution. In addition, when positively charged AgNPs come into contact with negatively charged microbial cells and proteins, they will adsorb each other, effectively attacking the cell walls and cell membranes, denaturing the cell proteins and preventing them from being metabolized and reproduced, so as to achieve the purpose of sterilization [34]. The film samples loaded with AgNPs possessing good protein adsorption properties is a particularly important point, as they can adsorb microorganisms to the outside of the dressing and utilize the AgNP bactericidal mechanism to play a highly effective antimicrobial effect, thereby avoiding to cause wound infections.

### 2.6. Optical Transparency

**Figure 6:**
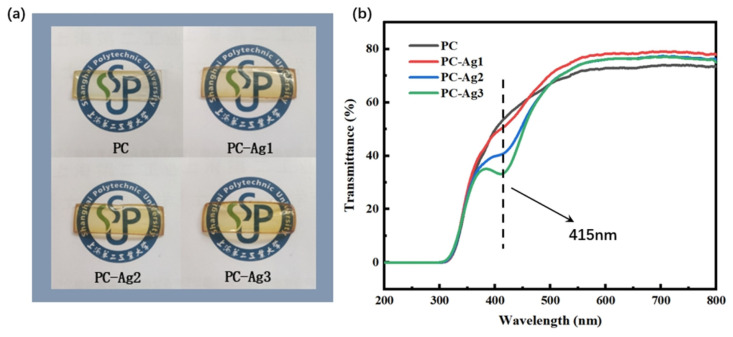
(**a**) Physical comparison of samples PC, PC-Ag1, PC-Ag2, and PC-Ag3; (**b**) UV transmittance results.

Normal dressings are usually not transparent and need to be changed frequently to visualize the healing status of the wound. For example, although the silver nanoparticle dressing prepared by Liu et al. [35] exhibits good swelling capacity, it is impossible to observe the wound condition from the outside. Meanwhile, although the silver nanoparticle antibacterial film dressing prepared by Ilyas Benkhira et al. [36] exhibits a certain degree of transparency, its transparency is poorer compared with that of the dressing in this study. Furthermore, the color of their dressing becomes darker as the reduction degree of silver nanoparticles increases. However, frequent changes will cause the wound to meet the external environment several times, destroying the sterile environment established by the silver-loaded dressing to rapidly promote wound healing. Therefore, it is necessary to develop a dressing with good transparency that facilitates the observation of wound conditions. The PVA hydrogel used in this experiment is milky white with poor transparency. In order to realize the routine observation of the wound area without removing the dressing and to maintain the sterile environment for a long period of time, we chose to add a glycerol solution to the polymer matrix, which can effectively increase the transparency and elasticity [37,38]. It can be seen from the physical images of the film samples in Figure 6a that the film samples prepared by adding 1.5 mL of glycerol solution with a concentration of 0.06 g/mL has good transparency, and the pattern covered under the film can be clearly and intuitively observed. However, AgNPs also affect the optical transparency of the samples to a certain extent. As shown in the transmittance curve of the film samples in Figure 6b, the transmittance of the PC film samples is 73.44%, which has the effect of improving the optical properties of the materials, since Ag^+^ is a fast conducting ion in many crystalline and amorphous materials [39]. Therefore, after adding silver nanoparticles to PC polymer, the transmittance reached 77.94% and the optical properties were improved. Since the synthesized AgNPs have their own color, the transmittance gradually decreases to 75.02% with the increasing content of AgNPs. However, overall, the transmittance of the silver-loaded thin film samples was kept above 75%, which is a high level. In addition, it is also observed from the transmittance curves that the addition of AgNPs to the PC polymer produces valleys at 415 nm, and their intensity continues to increase with the increase in the content of doped AgNPs. Materials exhibit a strong absorption or scattering behavior upon interaction between electromagnetic radiation and conduction electrons, which is called the surface plasmon resonance effect (SPR) [40]. However, this new valley is attributed to the formation of charge transfer complexes; the appearance of this valley in the visible region is due to the SPR nature of the silver nanoparticles embedded in the PVA polymer dielectric.

### 2.7. Drug Loading and Release Properties

**Figure 7:**
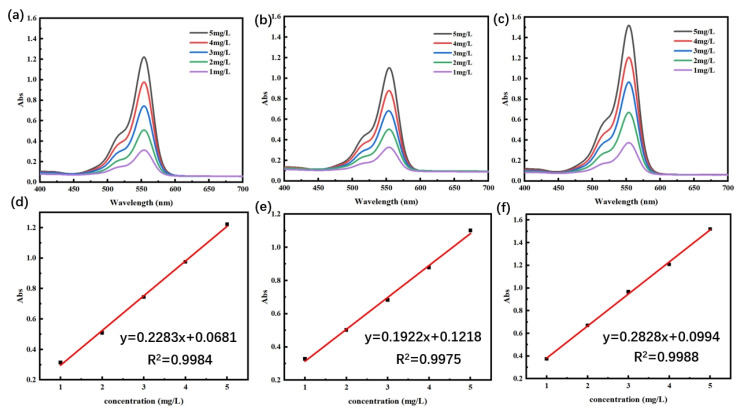
(**a**–**c**) The UV–Vis absorption spectra of RhB at three pHs (5.4, 7.4, and 8.2) and (**d**–**f**) the standard curves of RhB at three pHs, respectively.

The experimental methods for drug loading and drug release refer to the research method by Meng et al. [41] The standard curves of RhB solution at different pHs were plotted according to the highest absorption peaks at different concentrations in the UV–Vis absorption spectra, as shown in Figure 7a–c. As can be seen from Figure 7d–f, at pH = 5.4, the standard curve based on the absorbance of different concentrations obtained a linear regression equation: y = 0.2283x + 0.0681, R^2^ = 0.9984; at pH = 7.4, the standard curve based on the absorbance of different concentrations obtained a linear regression equation: y = 0.1922x + 0.1218, R^2^ = 0.9975; at pH = 8.2, the linear regression equations were obtained from the standard curves based on the absorbance at different concentrations: y = 0.2828x + 0.0994, R^2^ = 0.9988. It was proved that the concentration of RhB solution at the three pH levels had a good linear relationship with the absorbance in the range of 1 mg/L~5 mg/L. The results are summarized as follows.

**Figure 8:**
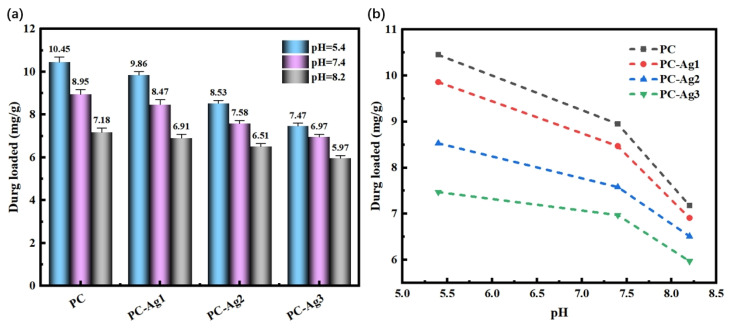
Drug loading of silver-loaded film samples: (**a**) different AgNP contents; (**b**) different pH buffer solutions.

As shown in Figure 8a, the drug loading of PC and PC-Ag with three different AgNP contents were 10.45 mg/g, 9.86 mg/g, 8.53 mg/g, and 7.47 mg/g, respectively, with an overall decreasing trend of drug loading at pH = 5.4; this pattern was still consistent at other pH conditions. Because AgNPs will occupy the pores of the hydrogel and reduce the porosity, and as the solubilization property is weakened, this leads to the decreasing trend of drug loading of PC-Ag with the increase in the AgNP content. As shown in Figure 8b, pH affects the drug loading of PC-Ag, which is significantly higher in acidic conditions than in alkaline conditions. This is again due to the ionization of the amino group of CS under acidic conditions, which makes the electrostatic repulsion increase, prompting the polymer chains inside the hydrogel polymer to increase the distance between them. As a result, the structure becomes loose, which facilitates the entry of solute molecules, improving the solubility properties, and making the drug more easily loaded in the hydrogel [42]. This is consistent with the research results of Sofia Alves et al. [43]: under acidic conditions, the protonation of chitosan chains increases and the electrostatic repulsion is strong, which leads to an increase in viscosity and chain entanglement. In addition, the ionization of the carboxyl group of rhodamine B under alkaline conditions, along with the decrease in the positive charge in CS and the increase in hydrogen bonding force between the hydrogel polymers, made the structure of the hydrogel film more compact. As a result, it was not easy for the solute molecules to penetrate into the inner part of the hydrogel, and the solubility property was weakened, so the ability of the drug loading was weakened as well.

**Figure 9:**
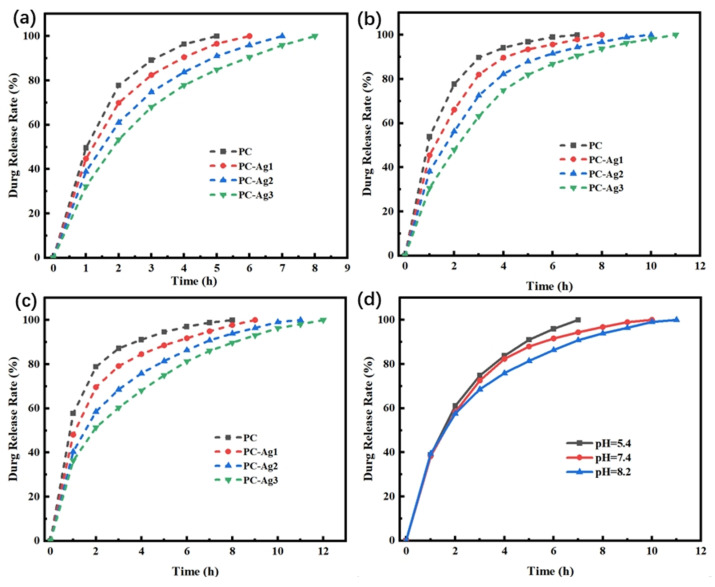
(**a**–**c**) Release rate profiles of model drug-carrying film samples with different AgNP contents in PBS buffer at pH = 5.4, 7.4, and 8.2; (**d**) release rate profiles of PC-Ag2 film samples in different pH buffers.

Regardless of the pH, PC-Ag undergoes explosive release within the first 2 h due to the concentration difference between the interior of the hydrogel and the release medium, after which the release curve tends to level off. Meanwhile, as shown in Figure 9a, the release rate of PC-Ag decreases with the increase in the AgNP content at pH = 5.4. The amount of drug release from the hydrogel is closely related to the internal structure: the larger the porosity of hydrogel, the faster the solute molecules penetrate, leading to the rapid expansion of the reticular structure, such that the drug can be released quickly. Since the addition of AgNPs will occupy the pores of the hydrogel and reduce the porosity, the hydrogel with AgNPs can have a better drug release. Moreover, pH = 7.4 and 8.2 is also consistent with this law.

As shown in Figure 9d, the same hydrogel samples first underwent explosive release at all three pHs, but the hydrogel samples under acidic conditions were completely released in 7 h, while the drug release curves under alkaline conditions were smoother and took longer to release. The reasons for this are, firstly, under alkaline conditions, the swelling property of the hydrogel is weak, it is not easy for the solute molecules from outside to enter into the interior of the hydrogel, and it is difficult for the loaded drug to diffuse rapidly into the release medium; in addition, the carboxyl group of rhodamine B is ionized with a negative charge, whereas CS belongs to the positively charged group, and the anion and cation combine and adsorb the rhodamine B in the interior of the hydrogel, which leads to a slow release rate in the alkaline conditions.

In summary, regardless of the pH value, the PC-Ag1 dressing exhibits the highest drug loading capacity, and the drug loading capacity under acidic conditions is higher than that under neutral and alkaline environments. However, the PC-Ag3 dressing shows the best sustained drug release effect under all environmental conditions, with a release duration of up to 12 h in an alkaline environment. The final drug release amount of all dressings can basically reach 90% of their respective drug loading capacities. Therefore, appropriate dressings can be selected based on the actual needs of the wound.

## 3. Conclusions

This study first reduced AgNPs (silver nanoparticles) via a redox method, then obtained a AgNP-loaded PVA/CS precursor solution through a simple one-pot method, and prepared silver-loaded hydrogel film dressings by drying them into films. The dried film samples exhibit excellent mechanical properties, showing high resistance to fracture under external forces, and possess good transparency, enabling intuitive observation of wound healing status. This feature avoids frequent dressing changes and reduces the risk of bacterial infection. Protein adsorption experiments demonstrate that the silver-loaded hydrogel film dressings have superior protein adsorption performance, which can adsorb microorganisms on the dressing surface. In addition, the dressing prepared by this method has a good transparency, up to 77.94%, which facilitates routine observation of the wound area without removing the dressing. The drug release capability of the film dressing demonstrates its practical application value and the provides necessary conditions for promoting wound healing. In summary, the PC-Ag film dressing combines ideal physicochemical properties, demonstrating significant clinical application potential as a new generation wound dressing.

## 4. Materials and Methods

### 4.1. Materials

Polyvinyl alcohol (PVA, analytical grade) was purchased from Sinopharm Chemical Reagent Co., Ltd. (Shanghai, China) Chitosan (CS, deacetylated degree ≥ 90%) was obtained from Shanghai Titan Technology Co., Ltd. (Shanghai, China). Acetic acid (analytical grade), silver nitrate (AgNO_3_, analytical grade), sodium citrate (SC, analytical grade), and glycerol were all supplied by Sinopharm Chemical Reagent Co., Ltd. Sodium borohydride (NaBH_4_, analytical grade; purity ≥ 96%) and glutaraldehyde (25 wt%) were purchased from Shanghai Titan Technology Co., Ltd. Bovine serum albumin (BSA, analytical grade) was obtained from Shanghai Titan Technology Co., Ltd.

### 4.2. Synthesis of Nanosilver

In the present experiment, nanosilver solutions were prepared by redox method. Different amounts of sodium borohydride and ultrapure water were taken in a beaker and mixed with stirring at room temperature until complete solution; different concentration gradients (4 µM–128 µM) of NaBH_4_ solution were prepared and kept aside. An amount of 17 mg of AgNO_3_, 80 mg of SC, and 110 mL of ultrapure water were taken in a beaker and mixed with stirring at 60 °C until complete dissolution. Keeping the temperature constant, the NaBH_4_ solution was added drop by drop to the AgNO_3_ solution containing SC under continuous stirring, and the solution was found to change gradually from colorless to brownish yellow. The successful synthesis of silver nanoparticles was proved by determining the absorbance of the samples in the UV–visible wavelength range of 200 nm to 800 nm using a UV–visible spectrophotometer (Shimadzu, Kyoto, Japan, UV 2600).

### 4.3. Preparation of Silver-Loaded Hydrogel Films

**Figure 10:**
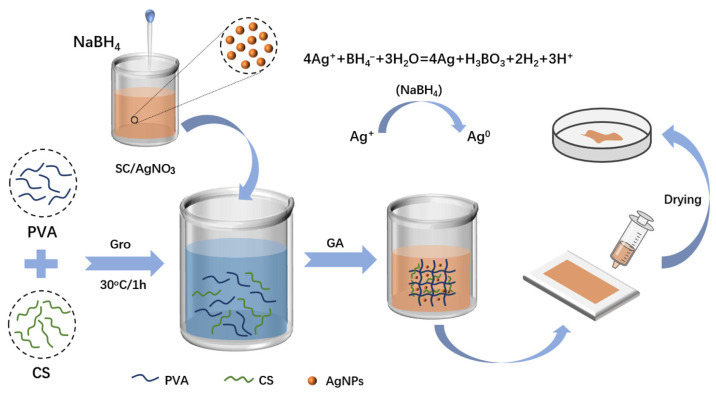
Schematic diagram of the process of preparing silver-loaded hydrogel film dressing.

The entire preparation process of silver-loaded hydrogel film samples is shown in Figure 7. In the figure, Gro stands for glycerol and GA stands for glutaraldehyde. A PVA solution of 10 wt% concentration was prepared by taking 2 g of PVA and ultrapure water in a beaker, and heating and stirring at 100 °C until PVA was completely dissolved. A CS solution with a concentration of 1 wt% was prepared by taking 0.1 g of CS and 2% acetic acid solution in a beaker, and stirring at room temperature until CS was completely dissolved. The 10 wt% PVA solution and 1 wt% CS solution were stirred at room temperature at a volume ratio of 2:1 until homogeneous; subsequently, 1.5 mL of glycerol solution with a concentration of 0.06 g/mL was added, and stirring was continued until thorough mixing was achieved. At room temperature, 0.5 mL, 1 mL, and 1.5 mL of silver nanoparticle (AgNP) solution were added to the PVA/CS mixed solution, respectively. According to the different amounts of AgNPs added, the samples were sequentially labeled as PC (with no AgNPs added), PC-Ag1 (with 0.5 mL of AgNP solution added), PC-Ag2 (with 1 mL of AgNP solution added), and PC-Ag3 (with 1.5 mL of AgNP solution added). After stirring to achieve uniform mixing, 1 mL glutaraldehyde solution was added for cross-linking; finally, the precursor solution was poured into a mold and placed in an oven at 50 °C for drying to form a film. After 24 h, the formed silver-loaded hydrogel film samples were removed from the mold and sealed for storage for later use. The preparation process of silver-loaded hydrogel film samples is shown in Figure 10.

### 4.4. Characterization

The prepared silver-loaded hydrogel film samples were sprayed with gold for 10 min, and the morphology structure and EDS elemental mapping pattern of the samples were observed using a scanning electron microscope with a scanning voltage of 10 KV. A Fourier transform infrared (FTIR) spectrometer (Nicolet, Waltham, MA, USA iS10) was used to examine the functional groups in the sample materials and to determine whether the desired substances were introduced into the system, and a KBr press sample preparation method was used with a scanning range of 4000–500 cm^−1^. XRD patterns of the thin film samples were determined using an X-ray diffractometer (Karlsruhe, Germany, D8-Advance). The aqueous components of the synthesized silver nanoparticles were sampled and the UV–Vis absorption spectra of the solutions were measured using a UV–Vis spectrophotometer (Shimadzu, UV 2600), and the size distribution and zeta potential values of the synthesized AgNPs were evaluated using a particle size and zeta potential analyzer (Malvern, UK, Zetasizer Advance).

### 4.5. Mechanical Testing

Tensile tests were performed on samples using a Shimadzu AGS-X universal material testing machine configured with a 1000 N tensile transducer. The samples were prepared into uniform strips with a length of 40 mm, width of 5 mm, and thickness of 2 mm, and tensile tests were conducted at a loading rate of 100 mm/min to analyze the mechanical properties of the film samples.

### 4.6. Swelling Rate and Porosity Performance

Swelling rate: After drying the sample, weigh it and record it as *W*_0_; then, immerse it in ultrapure water, and take it out and wipe off the water after *t* time, and weigh it and record it as *W_t_* (every 2 h) until it reaches the dissolution equilibrium state. The formula for calculating the swelling rate is shown in Equation (1).

(1) $$SR_{t}=\frac{W_{t}-W_{0}}{W_{0}}*100\%$$

Porosity: The porosity of the film samples was analyzed using the solution displacement method. First, the samples were placed in a vacuum drying oven at 60 °C to remove the water, weighed, and recorded as *m*_1_. Meanwhile, the volume *V* of the dry gel was measured; then, the dry gel was submerged in a beaker containing the same amount of anhydrous ethanol, and the vacuum was drawn until the dry gel sank to the bottom. Following this, the dry gel was taken out after 48 h, and was weighed and recorded as *m*_2_, and the porosity was calculated (density of ethanol: 0.7893 g/cm^3^). The porosity calculation formula is shown in Equation (2).

(2) $$P(\%)=\frac{m_{2}-m_{1}}{\rho V}*100\%$$

### 4.7. Adsorption Properties

The protein adsorption properties of the film samples were determined using bovine serum albumin (BSA). The samples were immersed in BSA solution (2 mg/mL) for 24 h. The absorbance of PC, PC-Ag1, PC-Ag2, and PC-Ag3 film samples adsorbed in BSA solution was determined at 280 nm.

### 4.8. Transparency Testing

As a hydrogel film wound dressing, having a high degree of transparency facilitates the observation of the degree of wound healing to determine the need to change the dressing or change the treatment plan. The transmittance of the film samples was measured using a UV–visible spectrophotometer to quantify the transparency. We then placed the blank substrate into the sample position of the sample holder, leaving the reference position empty, and set the transmittance at this point as 100%. Wear dust-free gloves to place the treated silver nanoparticle thin film dressing into the sample position of the sample holder, ensuring that the thin film completely covers the light path and that the sample surface is not tilted. After confirming that the sample is placed correctly, start the scanning process. To ensure data reliability, test at least 3 samples from different positions in the same batch, and repeat the scanning 3 times for each sample. The average value shall be taken as the final data.

### 4.9. Drug Loading and Releasing Test

During the preparation of the RhB hydrogel for model drug loading, the mass of the dry gel *M* was noted; after removing the gel dissolved and loaded with the drug, the residual RhB solution was collected and its volume was measured and noted as *V*. The absorbance of the residual RhB solution was determined by UV–visible absorption spectroscopy, and the concentration of RhB solution then was calculated from the standard curve of the RhB corresponding to the original standard concentration, and noted as *C*. Finally, the amount of drug loading in the sample was quantitatively calculated according to the drug loading formula. Finally, the drug loading of the sample in 24 h was quantitatively calculated according to the drug loading formula. The drug loading (*DL*) formula is shown in Equation (3), where *C*_0_: initial concentration of RhB solution; *V*_0_: initial volume of RhB solution; *C*: concentration of RhB solution remaining after loading drug; *V*: volume of RhB solution remaining after loading drug.

(3) $$\mathrm{DL}=\frac{C_{0}V_{0}-\mathrm{CV}}{M}$$

The obtained model drug-loaded hydrogel samples were placed in 10 mL beakers containing PBS buffer of different pH levels (pH = 5.4, 7.4, and 8.2) and stored in a sealed condition. The appropriate amount of solution was placed into the cuvette every 1 h, and the same amount of PBS buffer was added to the beaker and continued to be stored in sealed condition. The solution in the cuvette was analyzed by ultraviolet–visible spectrophotometer for ultraviolet absorption spectroscopy; its absorbance at 553 nm was determined, and the absorbance of the solution was measured every 1 h until the absorbance stabilized, which indicated that the drug had been completely released at this time. The concentration of the solution at this time can be calculated according to the standard curve of RhB, but the concentration is proportional to the cumulative release rate while the concentration is also proportional to the absorbance, so the curve of the drug release rate can be plotted according to the relationship of the change in absorbance.

## Data Availability

The data supporting the findings of this manuscript are available from the corresponding authors upon reasonable request.
